# Chain-End Functionalization of Poly(ε-caprolactone) for Chemical Binding with Gelatin: Binary Electrospun Scaffolds with Improved Physico-Mechanical Characteristics and Cell Adhesive Properties

**DOI:** 10.3390/polym14194203

**Published:** 2022-10-07

**Authors:** Ilya Nifant’ev, Victoria Besprozvannykh, Andrey Shlyakhtin, Alexander Tavtorkin, Sergei Legkov, Maria Chinova, Irina Arutyunyan, Anna Soboleva, Timur Fatkhudinov, Pavel Ivchenko

**Affiliations:** 1 A.V. Topchiev Institute of Petrochemical Synthesis RAS, 29 Leninsky Pr., 119991 Moscow, Russia; 2 Chemistry Department, M.V. Lomonosov Moscow State University, 1–3 Leninskie Gory, 119991 Moscow, Russia; 3 Chemistry Department, National Research University Higher School of Economics, 20 Miasnitskaya Street, 101000 Moscow, Russia; 4 Research Center for Obstetrics, Gynecology and Perinatology, Ministry of Healthcare of the Russian Federation, 4 Oparin Street, 117997 Moscow, Russia; 5 Institute of Medicine, Peoples’ Friendship University of Russia, Miklukho-Maklaya 6 Street, 117198 Moscow, Russia; 6 Research Institute of Human Morphology, 3 Tsyurupy Street, 117418 Moscow, Russia

**Keywords:** electrospinning, gelatin, *N*-hydroxysuccinimide, poly(ε-caprolactone), ring-opening polymerization, cell adhesion, physico-mechanical characteristics

## Abstract

Composite biocompatible scaffolds, obtained using the electrospinning (ES) technique, are highly promising for biomedical application thanks to their high surface area, porosity, adjustable fiber diameter, and permeability. However, the combination of synthetic biodegradable (such as poly(ε-caprolactone) PCL) and natural (such as gelatin Gt) polymers is complicated by the problem of low compatibility of the components. Previously, this problem was solved by PCL grafting and/or Gt cross-linking after ES molding. In the present study, composite fibrous scaffolds consisting of PCL and Gt were fabricated by the electrospinning (ES) method using non-functionalized **PCL1** or NHS-functionalized **PCL2** and hexafluoroisopropanol as a solvent. To provide covalent binding between **PCL2** and Gt macromolecules, NHS-functionalized methyl glutarate was synthesized and studied in model reactions with components of spinning solution. It was found that selective formation of amide bonds, which provide complete covalent bonding of Gt in PCL/Gt composite, requires the presence of weak acid. With the use of the optimized ES method, fibrous mats with different PCL/Gt ratios were prepared. The sample morphology (SEM), hydrolytic resistance (FT-IR), cell adhesion and viability (MTT assay), cell penetration (fluorescent microscopy), and mechanical characteristics of the samples were studied. **PCL2**-based films with a Gt content of 20 wt% have demonstrated the best set of properties.

## 1. Introduction

Electrospinning (ES) technology for preparing biodegradable polymer fibers has attracted extensive attention owing to a large specific surface area, high porosity, controllable fiber diameter, and good gas permeability of the materials obtained [1,2,3,4,5]. ES fiber mats can be widely used in tissue engineering, wound dressing, drug delivery systems, skin care, and other biomedical applications [3,4,6,7,8,9,10,11,12,13,14]. The properties of the ES fiber mats, such as hydrophilicity, biocompatibility, biodegradability, cell interaction, and mechanical strength, are dependent on the chemical nature of the materials. Poor mechanical stability of natural polymers and inferior biocompatibility of synthetic polymers may impede progress towards the optimal set of characteristics of the ES scaffolds. These shortcomings can be mitigated through the use of the composition of natural and synthetic polymers. It is expected that synergy of the useful features of synthetic and natural polymers can be achieved through efficient compatibilization of the components and precise control of the components’ ratio.

**Scheme 1:**
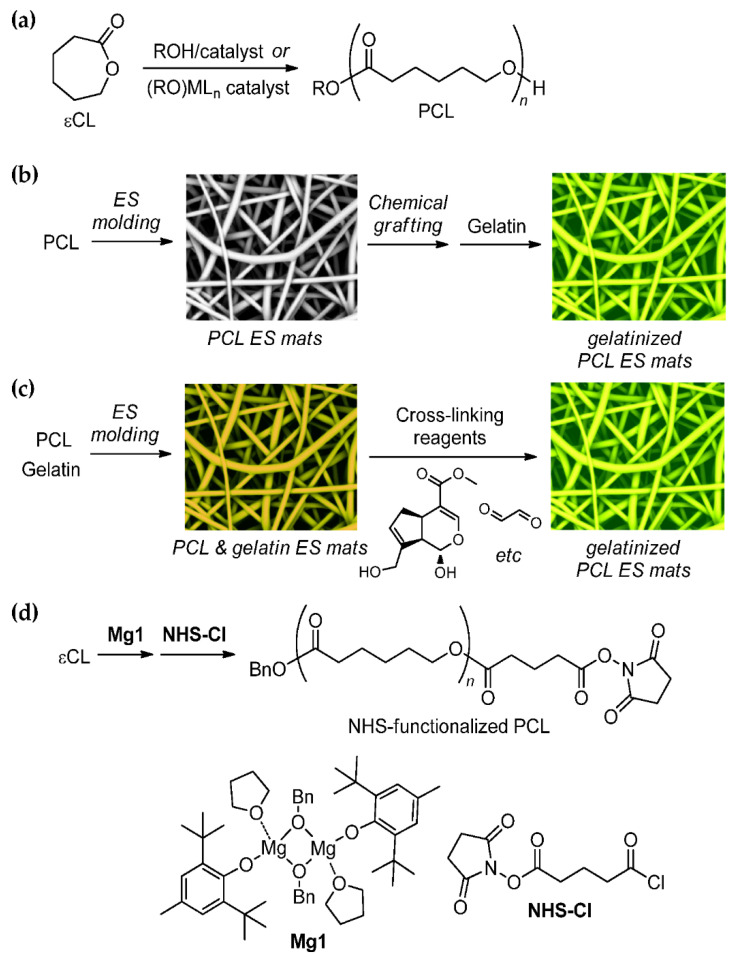
(**a**) Catalytic ROP of εCL; (**b**,**c**) actual methods of the obtaining of PCL/gelatin ES scaffolds with gelatin covalently bound to PCL and cross-linked gelatin, respectively; (**d**) the synthesis of NHS-functionalized PCL [19].

Among synthetic biocompatible polymers, poly(ε-caprolactone) (PCL)-based ES materials are widely studied [15], largely owing to the approval of PCL by the Food and Drug Administration [16]. The synthesis of PCL is based on the ring-opening polymerization (ROP) of ε-caprolactone (εCL) (Scheme 1a), allowing to obtain εCL (co)polymers with given microstructure and molecular weight characteristics [17,18].

The absorption time of PCL in vivo is more than 2 years [20]; poor hydrophilicity of PCL reduces cell adhesion. Therefore, it would be desirable to blend or copolymerize PCL with other hydrophilic and biocompatible polymers. Gelatin (Gt) is a hydrophilic and non-immunogenic natural-origin polymer that promotes cell adhesion, differentiation, and proliferation [21]. PCL/Gt-based ES composite nano- and microfibers seem to be promising for different biomedical applications [21,22,23,24,25,26,27,28,29,30,31,32,33,34,35], and the studies of PCL/Gt ES scaffolds have intensified significantly in recent years [36,37,38,39,40,41,42,43,44,45,46,47,48,49,50,51,52,53].

Limited compatibility of hydrophobic PCL and hydrophilic water-soluble Gt entails the following problems: the deterioration of the physico-mechanical properties of the ES films and washing off the Gt after a short period of maintenance in aqueous physiological media. Gt contains ~3 wt% of lysine [54], which gives an opportunity for cross-linking between Gt macromolecules or between PCL and Gt macromolecules. Such cross-linking may be performed before and after ES. In early and following studies, preparation of PCL/Gt ES films was accomplished by chemical grafting of the PCL fibers, followed by the treatment with Gt [22,23,24,44,52] (Scheme 1b). The task of the ‘stabilization’ of Gt in binary ES scaffolds was also solved via the treatment of PCL/Gt ES films by gelatin cross-linker reagents such as glutaraldehyde [28,45], genipin [26,30,42] (Scheme 1c), and other active organic compounds [29,40,42,55], employing recent progress in cross-linking of ES Gt nanofibers [56]. In this way, known methods of the preparation of PCL/Gt ES scaffolds with chemically bonded Gt have used chemical linking between macromolecules *after* ES molding.

However, it is also possible to prepare PCL/Gt ES solution containing functionalized PCL macromolecules that are able to react with Gt amino groups, thus providing chemical binding between PCL and Gt macromolecules *before* ES molding. We recently developed an efficient method for the chain-end functionalization of polyesters via termination of the [(BHT)Mg(μ-OBn)(THF)]_2_ (**Mg1**, Scheme 1d) catalyzed ‘living’ ROP by NHS-substituted acyl chlorides and have shown that, when using 2,5-dioxopyrrolidin-1-yl 5-chloro-5-oxopentanoate (**NHS-Cl**, Scheme 1d), the best degree of the functionalization of PCL was achieved [19].

In the present work, we synthesized PCL and NHS-functionalized PCL, demonstrated the effectiveness of NHS functionalization for the further chemical bonding with Gt, prepared PCL/Gt ES films, and conducted a comparative study of the effect of NHS functionalization of PCL on the characteristics of the fibrous materials obtained.

## 2. Materials and Methods

### 2.1. Solvents and Reagents

Most of the solvents and chemicals were supplied by Merck (Darmstadt, Germany). Toluene (99.5%), triethylamine (≥99%), diethyl ether (Et_2_O, ≥99%), and tetrahydrofuran (THF, ≥99%) were refluxed over sodium/benzophenone ketyl and distilled. Ethanol (EtOH, ≥99%) and methanol (MeOH, ≥99%) were refluxed over Mg turnings (≥99.8%) and distilled. εCL and benzyl alcohol were distilled under reduced pressure and stored in an argon atmosphere. *N*-hydroxysuccinimide, glutaric anhydride, ethyl acetate (EtOAc, ≥99%), NaHCO_3_, oxalyl chloride, di-*n*-butylmagnesium (1M solution in *h*-heptane), 2,6-di-*tert*-butyl-4-methylphenol, *N*,*N*-dimethylpyridin-4-amine (DMAP), Gt Type A (from porcine skin, gel strength 300 g Bloom), and dimethyl sulfoxide (DMSO) were used as purchased. 1,1,1,3,3,3-Hexafluoropropan-2-ol (HFIP, ≥99%) was supplied by P&M Invest (Moscow, Russian Federation) and used without further purification.

The catalyst [(BHT)Mg(μ-OBn)(THF)]_2_ [57] and 2,5-dioxopyrrolidin-1-yl 5-chloro-5-oxopentanoate (**NHS-Cl**) [19,58] were synthesized according to the described methods. 2,5-Dioxopyrrolidin-1-yl methyl glutarate (**NHS-OMe**), obtained previously from methyl glutarate [59], was prepared by us via methanolysis of **SIG-Cl**; the protocol for the synthesis and NMR spectra of **NHS-OMe** are presented in Section S1 in the Supplementary Materials.

### 2.2. Instruments and Methods

The ^1^H and ^13^C {^1^H} NMR spectra were recorded on a Bruker AVANCE 400 spectrometer (400 MHz, Bruker Corporation, Billerica, MS, USA) at 20 °C. CDCl_3_ (D 99.8%, Cambridge Isotope Laboratories, Inc., Tewksbury, MS, USA) was used as purchased. The chemical shifts were reported relative to the solvent residual peaks (*δ* = 7.26 and 77.0 ppm for ^1^H and ^13^C {^1^H} NMR spectra, respectively).

Size exclusion chromatography (SEC) measurements were performed in THF (40 °C, flow rate 1 mL·min^−1^) on 1260 Infinity II (Agilent Technologies, Santa Clara, CA, USA) integrated instrument equipped with a PLgel MIXED-C column (2 × 10^2^–2 × 10^6^ Da), an autosampler, and a refractive index detector. The measurements of the number average molar mass (*M*_n_) and mass average molar mass (*M*_w_) were recorded with universal calibration according to a polystyrene standard; polymer dispersity (*Đ*_M_) was determined as the ratio of *M*_w_/*M*_n_.

SEM images were obtained using a Phenom XL microscope (Thermo Fisher Scientific, Waltham, MA, USA) at an accelerating voltage of 5.0 kV.

Huber MPC-E immersion thermostat (Huber Kältemaschinenbau, Offenburg, Germany) was used in experiments on hydrolytic degradation of the ES films in distilled water, PBS (0.1 M, LLC ‘Pushchino Laboratories Company’, Pushchino, Russian Federation), and NaHCO_3_ (0.1 M). The temperature of hydrolysis was 37 °C. After being removed from the media, the samples were pre-dried by blotting paper, dried in vacuo, and analyzed using Fourier transform infrared (FT-IR) spectroscopy.

FT-IR spectra were recorded on IFS 66v/S spectrometer equipped with a DLaTGS detector (Bruker, Billerica, MA, USA). The following experimental parameters were used: attenuated total reflection method, ZnSe crystal, spectral range 600–4000 cm^−1^, resolution 2 cm^−1^, and 15 scans. The FT-IR spectra of the samples are presented in Section 3.3.2 in the main text and in Section S3 in the Supplementary Materials.

The ES films of 25 mm length, 5 mm wide, and 0.5 mm thick were mechanically tested using an EZ-Test EZ-SX universal tensile testing machine (Shimadzu Corp., Kyoto, Japan). The experimental control was carried out by TRAPEZIUM X software (Shimadzu Corp., Kyoto, Japan); the tensile speed was 1 mm·min^−1^. Young’s modulus was defined as the slope of the linear part of the stress–strain curve.

### 2.3. Synthesis of Poly(ε-caprolactone) and NHS-Functionalized Poly(ε-caprolactone)

#### 2.3.1. Poly(ε-caprolactone) PCL1

A preheated 100 mL glass ampoule was equipped with a magnetic stir bar; 10.0 g of εCL (87.6 mmol, 9.71 mL, 100 eq.) was placed into the ampule and then the ampoule was filled with dry argon and closed with a septum. THF (34 mL) was added (to make a resulting concentration of monomer of approximately 2M), then the reaction mixture was cooled to 5 °C, and a solution of **Mg1** (372 mg, 0.88 mmol, 1 eq.) in THF (3 mL) was added. After 4 h of stirring at 20 °C, AcOH (100 μL) was added and the obtained polymer solution was evaporated under reduced pressure and redissolved in dichloromethane (100 mL). The solution was washed with solution of 5 eq. of 8-hydroxyquinoline in 0.7 M HCl acid (2 × 20 mL), then with 0.7 M HCl (2 × 20 mL), and finally with water (2 × 50 mL). The polymer was precipitated in Et_2_O, filtered, and dried in vacuo using argon-vacuum line equipped with RZ 6 rotary pump (Vacuubrand GMBH, Wertheim, Germany). The yield was 8.3 g (83%). The ^1^H NMR spectrum of the homopolymer **PCL1** is presented in Figure S3 in the Supplementary Materials.

#### 2.3.2. NHS-Functionalized Poly(ε-caprolactone) PCL2

ROP of εCL was conducted with the same loads of the monomer, solvents, and **Mg1**. After 4 h of stirring at 20 °C, a solution of **NHS-Cl** (654 mg, 2.64 mmol, 3 eq.) in THF (3 mL) was added. The reaction mixture was stirred for an additional 4 h, evaporated under reduced pressure, and redissolved in dichloromethane (100 mL). The solution was washed with a solution of 5 eq. of 8-hydroxyquinoline in 0.7 M HCl acid (2 × 20 mL), then with 0.7 M HCl (2 × 20 mL), and finally with water (2 × 50 mL). The polymer was precipitated in Et_2_O and dried in vacuo using argon-vacuum line equipped with RZ 6 rotary pump (Vacuubrand GMBH, Wertheim, Germany). The yield was 7.7 g (77%). The ^1^H NMR spectrum of the functionalized polymer **PCL2** is presented in Figure S4 in the Supplementary Materials.

### 2.4. Preliminary Experiments on ES Molding and Model Experiments on Reactivity of NHS-OMe

#### 2.4.1. ES Molding of PCL2/Gt Mixtures

A series of experiments were conducted with the use of HFIP as a solvent to achieve the best morphology of ES fibers. For example, Gt (0.30 g) and polymer **PCL2** (0.70 g) were dissolved in HFIP (3 mL). After 2 days, HFIP (2 mL) was added and the obtained solution was electrospun at a flow rate of 0.8 mL·h^−1^ using a 5 mL syringe with a spinneret (0.8 mm diameter needle) and a collector (5 × 5 cm aluminum foil). The distance between spinneret and collector was 24 cm and the potentials were −4 kV (needle) and 16 kV (foil), while the amplitude was 2 cm. As a result, the sample of the ES film **ESf0** was obtained (see Figure S5 in the Supplementary Materials). This sample was subjected to hydrolytic degradation and studied by FT-IR spectroscopy (see Section 3.1.1).

#### 2.4.2. Model Experiments with NHS-OMe

HFIP (500 μL), CDCl_3_ (150 μL), and **NHS-OMe** (20 mg) were placed into a standard 5 mm NMR tube. The calculated amount of the reagent (Et_3_N, BuNH_2_, AcOH, py) was added and NMR spectra of the reaction mixture were registered at specified intervals.

### 2.5. Preparation of ES Films for Hydrolytic and Cell Culture Studies

#### 2.5.1. Preparation of **PCL1** ES film

A polymer **PCL1** sample (1 g) was dissolved in mixture of CHCl_3_ and MeOH (9:1 volume ratio, 3 mL). The solution was electrospun at a flow rate of 0.8 mL·h^−1^ using a 5 mL syringe with spinneret (0.8 mm diameter needle) and a collector (5 × 5 cm aluminum foil). The distance between the spinneret and collector was 24 cm and the potentials were −5 kV (needle) and 20 kV (foil). The back and forth motion of the collector was driven by a stepping motor (speed 5 mm·s^−1^, amplitude 2 cm). The sample **ESf1** was obtained.

#### 2.5.2. Preparation of **PCL1**/Gt ES Films

Gt (0.20 g) was dissolved in HFIP (5 mL), and AcOH (20 μL) and polymer **PCL1** (0.80 g) were added. The obtained solution was electrospun at a flow rate of 0.8 mL·h^−1^ using a 5 mL syringe with a spinneret (0.8 mm diameter needle) and a collector (5 × 5 cm aluminum foil). The distance between the spinneret and collector was 24 cm and the potentials were −10 kV (needle) and 40 kV (foil), while the amplitude was 4 cm. As a result, the sample of the ES film **ESf2** was obtained. The sample **ESf3** was obtained in the same manner using 0.30 g of Gt and 0.70 g of **PCL1**; the optimized potentials were −5 kV (needle) and 20 kV (foil).

#### 2.5.3. Preparation of **PCL2**/Gt ES Films

Gt (0.20 g) was dissolved in HFIP (3 mL), and AcOH (20 μL) and polymer **PCL2** (0.80 g) were added. After 2 days, HFIP (2 mL) was added and the obtained solution was electrospun at a flow rate of 0.8 mL·h^−1^ using a 5 mL syringe with a spinneret (0.8 mm diameter needle) and a collector (5 × 5 cm aluminum foil). The distance between the spinneret and collector was 24 cm and the potentials were −4 kV (needle) and 16 kV (foil); the amplitude was 2 cm. As a result, the sample of the ES film **ESf4** was obtained. The sample **ESf5** was obtained in the same manner using 0.30 g of Gt and 0.70 g of **PCL2**, while the optimized potentials were −4 kV (needle) and 16 kV (foil); the amplitude was 4 cm. The sample **ESf6** with 50 wt% content of Gt was prepared similarly to the sample **ESf5**.

### 2.6. In Vitro Experiments

#### 2.6.1. Cell Culture

Based on the data compiled and reported earlier [60,61], umbilical-cord-derived mesenchymal stem cells (UC-MSCs) were isolated using the mixed enzymatic-explant method from Wharton’s jelly of umbilical cord. Collection of umbilical cords was approved by the Commission of Biomedical Ethics at National Medical Research Center for Obstetrics, Gynecology, and Perinatology of the Ministry of Healthcare of Russian Federation, Moscow (Ethic’s committee approval protocol No. 12, 17 November 2016). Written informed consent was obtained from all participants prior to the study.

UC-MSCs were cultured in Dulbecco’s modified Eagle medium/F-12 (DMEM-F12) (PanEco, Moscow, Russian Federation) containing 10% fetal bovine serum (FBS; Thermo Fisher Scientific, Waltham, MA, USA) and 1% penicillin-streptomycin (PanEco, Moscow, Russian Federation) at 37 °C under 5% CO_2_ humidified atmosphere. UC-MSCs were detached from the culture substrate with a trypsin-EDTA solution (PanEco, Moscow, Russian Federation), then the cell count and viability were estimated using a TC20 Automated Cell Counter (Bio-Rad Laboratories, Inc., Hercules, CA USA).

#### 2.6.2. Cell Labeling

For cell visualization and cell count on the surface of polymer films, UC-MSCs were labeled with fluorescent red-orange vital dye PKH26 (Merck, Darmstadt, Germany) before seeding the scaffolds according to the manufacturer’s protocol. This protocol uses proprietary membrane labeling technology to incorporate a yellow-orange fluorescent dye with long aliphatic tails (PKH26) into lipid regions of the cell membrane [62]. The appearance of labeled cells may vary from bright and uniform to punctate or patchy, depending on the cell type being labeled and the extent to which membrane internalization occurs after labeling [63]. Cell nuclei were counterstained by Hoechst 33,342 (5 mg·mL^−1^, ThermoFisher Scientific, Waltham, MA, USA) within 10 min.

The observations were carried out with the use of a Leica DM 4000 B fluorescent microscope and LAS AF v.3.1.0 build 8587 software (Leica Microsystems GmbH, Wetzlar, Germany).

#### 2.6.3. Dynamic Cell Seeding of Scaffolds

The scaffolds were transferred into bioreactor tubes (SPL Life Sciences Co., Ltd., Gyeonggi-do, Korea) containing cell suspension (10 mL, 10^5^ cells·mL^−1^). Bioreactor tubes were fixed in an orbital shaker (BioSan, Riga, Latvia), placed in a CO_2_ incubator. Cell cultivation was carried out over 24 h at 70 rpm. The cell suspension was changed to freshly prepared; after 24 h of shaking, the seeded scaffolds were removed and used in the following experiments.

#### 2.6.4. MTT Assay

A quantitative assessment of cell seeding efficiency was performed using the MTT tetrazolium salt colorimetric assay. MTT (Merck, Darmstadt, Germany) was added to wells containing seeded scaffolds (disc of diameter 5 mm and thickness 0.5 mm) to a final concentration of 0.5 mg·mL^−1^. After 4 h of incubation, formazan was eluted from cells using DMSO within 30 min. After the formazan crystals had dissolved, the absorbance was determined spectrophotometrically at 570 nm using a reference wavelength of 630 nm on a microplate reader Multiskan GO Spectrophotometer (ThermoFisher Scientific, Waltham, MA, USA).

#### 2.6.5. Cryosectioning

To study cell penetration into fibrous mats, the cell-seeded scaffolds were embedded in Tissue-Tek^®^ O.C.T. Compound (Sakura Finetek Japan Co., Ltd., Tokyo, Japan) and frozen at −70 °C. Cross sections of 7 μm thick were made using cryotome Leica CM1900 (Leica Microsystems GmbH, Wetzlar, Germany) and SuperFrost slides (ThermoFisher Scientific, Waltham, MA, USA).

### 2.7. Statistical Analysis

The data are presented as the mean  ±  standard deviation (SD) and median with interquartile range. The data were analyzed by SigmaStat 3.5 software (Systat Software Inc., San Jose, CA, USA). For multiple comparisons in the case of a normal distribution of data, one-factor analysis of variance (one-way ANOVA) was used; in a case other than normal distribution, the Kruskal–Wallis criterion (ANOVA on ranks) was used. Values of *p* < 0.05 were considered statistically significant.

## 3. Results and Discussion

### 3.1. The Synthesis of PCL and Preliminary ES Molding Experiments

#### 3.1.1. The Synthesis of PCL1 and PCL2

The polymer samples **PCL1** and **PCL2** were obtained by ROP of εCL in THF media, where the complex **Mg1** (Scheme 1d) was used as a catalyst. As **Mg1**-catalyzed ROP of εCL is a ‘living’ process [57,64], degree of polymerization *DP*_n_ values were determined by the integration of the signals of BnO– end groups (δ 7.35 and 5.11 ppm) and PCL (δ 4.06, 2.30, 1.65, and 1.38 ppm) in ^1^H NMR spectra of the polymers (Figures S3 and S4 in the Supplementary Materials), and were equal to 129 and 135 for **PCL1** and **PCL2**, respectively. These values are relative to *M*_n_ of 14.8 and 15.5 kDa, which correlates with the SEC data (*M*_n_ = 16.0 and 16.8 kDa and *Đ*_M_ = 1.18 and 1.23 for **PCL1** and **PCL2**, respectively).

The degree of NHS functionalization in **PCL2** was determined by the integration of the characteristic signals of BnO–, –(CH_2_)_2_– succinimide fragment (δ 2.84 ppm), and –(CH_2_)_3_– glutarate fragment (δ 2.70, 2.36, and 2.06 ppm, respectively); ~65% of the –OH end-groups were found to be functionalized after termination of ROP by **NHS-Cl**. We considered this degree of functionalization as sufficient for the further experiments.

#### 3.1.2. Preliminary ES Molding Experiments and Hydrolytic Stability of PCL/Gt Composites

In recent publications, two types of solvents have been used for ES of PCL/Gt composites, fluorinated alcohols [29,30,31,33,35,36,39,41,43,45,49,51] and carboxylic acids (AcOH, HCOOH) [32,34,37,38,40,42,46,47,48,50,65]. In the present study, we set ourselves the task to provide rather high (but not excessive) chemical bonding between NHS-functionalized PCL and Gt macromolecules as a result of the reaction of NHS-terminated PCL with amino groups of Gt. We reasonably suggested that acidic solvents (AcOH or AcOH/HCOOH) are unusable for our purposes because of the protonation of the amino groups. Consequently, we chose fluorinated alcohol, HFIP, as a solvent for ES. To provide the interaction between **PCL2** and Gt, spinning solutions were kept for 2 days before molding. In the dozens of experiments with **PCL2** and Gt solutions, close to optimal concentrations and conditions were found (for the example of **ESf0**, see Section 2.4.1 and Figure S5 in the Supplementary Materials).

To evaluate the effectiveness of the covalent binding between **PCL2** and Gt, we carried out experiments on the hydrolytic degradation of the sample **ESf0** (Section 2.4.1) in neutral (distilled H_2_O, 0.1M PBS) and weakly basic (0.1M NaHCO_3_) media, which should be accompanied by dissolution of the unconjugated Gt. The use of weighting for such an estimation is not feasible owing to the high hydrophilicity of Gt, and we used FT-IR spectroscopy for comparison of the characteristic spectral lines of PCL and Gt (for more details, see Section 3.3.2). We found (see Figure S6 in the Supplementary Materials) that 7-day immersion of **ESf0** in aqueous media results in substantial loss of the Gt fraction, which indicates only partial binding between PCL and Gt. We proposed that this may be due to side reactions of NHS-terminated PCL with the components of the spinning solution, and carried out a separate study to clarify the issue.

### 3.2. The Study of the Reactivity of **NHS-OMe**

#### 3.2.1. The Synthesis of **NHS-OMe**

**Scheme 2:**
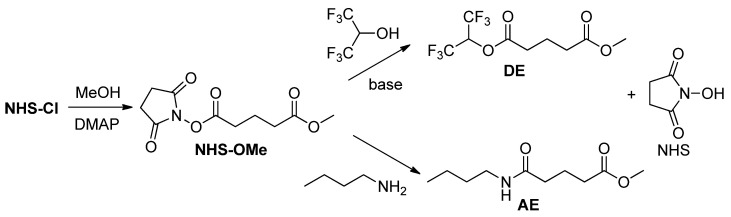
The synthesis of **NHS-OMe** and its reactivity in a model spinning solution.

**Figure 1:**
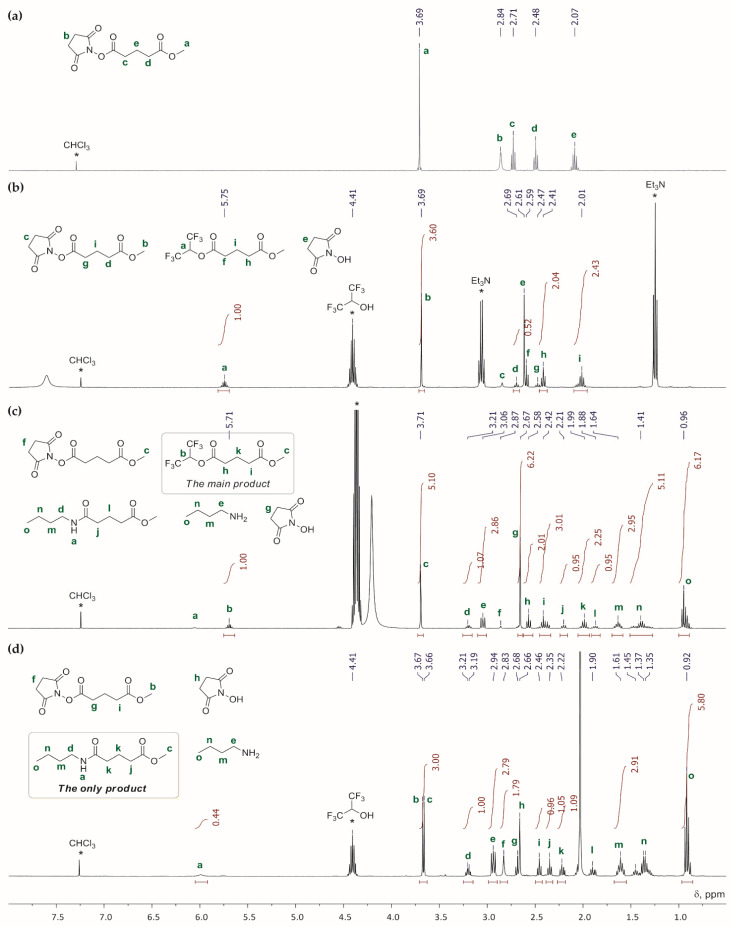
^1^H NMR spectra (400 MHz, CDCl_3_, 20 °C) of (**a**) **NHS-OMe** (reference); (**b**) **NHS-OMe** + HFIP in the presence of Et_3_N after 1 h; (**c**) **NHS-OMe** + HFIP + *n*-BuNH_2_ after 3 h; and (**d**) **NHS-OMe** + HFIP + *n*-BuNH_2_ in the presence of AcOH after 3 h.

To study the chemical behavior of NHS-functionalized PCL, we synthesized the model compound **NHS-OMe** (Scheme 2). Until quite recently, **NHS-OMe** was an undescribed compound. In 2022, **NHS-OMe** was prepared by Sessler and coll. [59] by the reaction of methyl glutarate with 1-ethyl-3-(3-dimethylaminopropyl)carbodiimide and *N*-hydroxysuccinimide followed by purification using flash chromatography; the yield was 90% (colorless liquid). Having chloroanhydride **NHS-Cl** at our disposal, we synthesized **NHS-OMe** by the methanolysis of **NHS-Cl** with the use of DMAP as a base (Scheme 2). The colorless reaction product (m.p. 38–39 °C, ^1^H NMR spectrum is presented in Figure 1a) was purified by crystallization from *n*-hexane. The lower yield of **NHS-OMe** (~70%) is partially offset by the simplicity of the method and by the availability of the reagents.

#### 3.2.2. The Reactivity of **NHS-OMe** in HFIP-Based Model Spinning Solution

First, we studied the chemical behavior of **NHS-OMe** (see Figure 1b for reference ^1^H NMR spectrum) in HFIP in the presence of bases. When using Et_3_N (Figure 1b), we detected fast formation of the corresponding hexafluoroisopropyl/methyl diester (compound **DE** in Scheme 2), and characteristic signals of **DE** appeared in the reaction mixture within a minute (see Figure S7 in the Supplementary Materials). This is understandable because of the relatively higher acidity of HFIP (pK_a_ = 9.3) [66] in comparison with Et_3_NH^+^ (pK_a_ = 10.8). Apparently, it is the basicity of the amine that affects the formation of **DE**, thus, for example, in the presence of pyridine (pK_a_ = 5.3), we did not observe esterification (see Figure S8 in the Supplementary Materials).

However, *n*-BuNH_2_, which can be considered as a model compound for reactive lysine fragments in Gt, has the same basicity (pK_a_ = 10.78) as Et_3_N. When conducting the reaction between **NHS-OMe** and *n*-BuNH_2_, we detected the formation of both diester **DE** and amidoester (compound **AE** in Scheme 2) in a ~1:1 ratio (Figure 1c). One would expect the similar behavior of NHS-functionalized **PCL2** in the solution of Gt in HFIP. Apparently, this was the reason for partial binding of Gt observed for the **ESf0** sample.

We proposed that the formation of diester **DE** can be inhibited by the addition of a minimal amount of the weak acid (for example, AcOH), and conducted the reaction of **NHS-OMe** with *n*-BuNH_2_ in HFIP in the presence of AcOH. During this reaction, only **AE** and NHS formed, and diester **DE** was not detected even in trace amounts (Figure 1d).

### 3.3. ES Molding and Hydrolytic Stability of the Composite Films

#### 3.3.1. ES Molding

Based on the results of the experiments with **NHS-OMe**, we concluded that **PCL2**/Gt spinning solution should contain a certain amount of AcOH to prevent the formation of the HFIP ester instead of chemical binding with Gt with a formation of an amide bond. This assumption was confirmed by the model reaction of Gt with **NHS-OMe** in HFIP in the presence of AcOH, which resulted in the selective formation of amide (see Figures S9 and S10 in the Supplementary Materials). Besides, as demonstrated previously by Zhang and coll., the introduction of a tiny amount of AcOH to the PCL/Gt solution in 2,2,2-trifluoroethanol prevents phase separation and enhances the morphology of the ES fibers [27].

**Figure 2:**
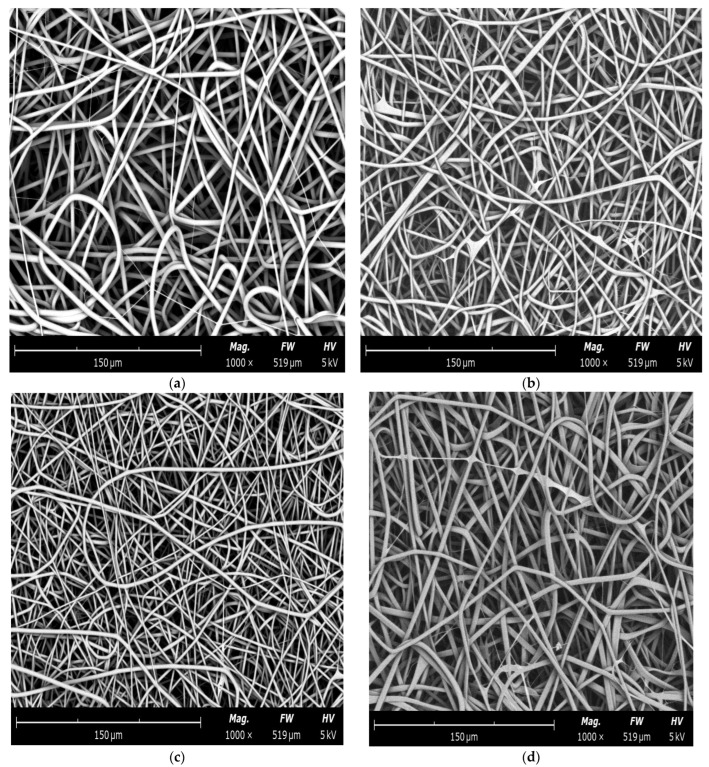
SEM images of the ES fibrous mats: (**a**) **ESf2**; (**b**) **ESf3**; (**c**) **ESf4**; and (**d**) **ESf5**.

In our experiments, we selected HFIP as a solvent and optimized the conditions of the ES molding for each type of the polymer and PCL/Gt ratio (see Section 2.5). The samples **ESf2** and **ESf3** with 80:20 and 70:30 PCL/Gt ratios, respectively, were prepared from **PCL1**, while the samples **ESf4** and **ESf5** with the same PCL/Gt ratios were prepared from NHS-functionalized **PCL2**. The sample **ESf6** with a 50:50 **PCL2**/Gt ratio was also obtained. When using HFIP for the preparation of the spinning solutions, we did not observe marked sedimentation; however, a minor positive impact of AcOH on the morphology of ES fibers was seen by comparing the microphotos of the ES mats **ESf0** obtained as a result of preliminary ES molding (Figure S5 in the Supplementary Materials) and PCL/Gt ES mats **ESf2**–**ESf5** (Figure 2).

It can also be seen that the samples **ESf2** and **ESf4** (20 wt% of Gt) had a more homogeneous morphology. An increase in the PCL/Gt ratio to 50/50 resulted in worsening of the morphology of the ES film (sample **ESf6**, Figure S11 in the Supplementary Materials).

#### 3.3.2. Hydrolytic Stability

**Figure 3:**
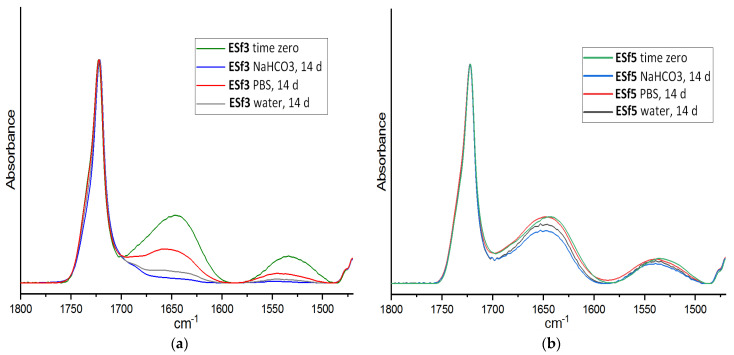
FT-IR spectra of the samples **ESf3** (**a**) and **ESf5** (**b**) before (green line) and after 14-day immersion in H_2_O, 0.1M NaHCO_3_, and 0.1M PBS (gray, blue, and red lines, respectively).

Since **ESf0** with 30 wt% Gt content was used in our preliminary experiments on biodegradation, the samples **ESf3** and **ESf5** were selected for the study of the binding strength of PCL and Gt macromolecules in composites. We conducted hydrolytic experiments at 37 °C in three reaction media: distilled water, 0.1M aq. PBS solution, and 0.1 M aq. NaHCO_3_ solution. After two weeks, the samples were removed from the solutions, dried, and studied by FT-IR spectroscopy for the determination of the residual Gt (Figure 3). Based on the literature data [25,34,35], we selected the spectral region of 1450–1800 cm^−1^ as a ‘fingerprint’ area that includes non-overlapping characteristic signals of PCL (1720 cm^−1^) and Gt (1530 and 1640 cm^−1^).

For the sample **ESf3** (non-functionalized **PCL1**), we observed almost full elimination of Gt after 14-day immersion in distilled water and 0.1 M aqueous NaHCO_3_ solution. Here, 0.1 M PBS solution turned out to be less ‘invasive’; however, at least half of Gt had been washed out.

For **PCL2**-based sample **ESf5**, we observed a more encouraging result; that is, after 14-day immersion, most of Gt remained bonded with PCL. Note that the PBS solution appeared to have no effect on the sample. In this way, **PCL2**-based composite with a PCL/Gt ratio of 70/30 mainly contained Gt, covalently bound to PCL macromolecule(s). However, reducing the PCL/Gt ratio to 50/50 (sample **ESf6**) resulted in obtaining less hydrolytically stable fibrous film (FT-IR spectrum is presented in Figure S12 in the Supplementary Materials). In neutral aqueous media (water, PBS), we observed substantial elimination of Gt, while in 0.1M NaHCO_3_, the sample fell apart. Based on the hydrolytic degradation test results, we selected the samples **ESf1** (**PCL1**, no Gt), **ESf2** (**PCL1**/Gt 80:20), **ESf3** (**PCL1**/Gt 70:30), **ESf4** (**PCL2**/Gt 80:20), and **ESf5** (**PCL2**/Gt 70:30) for further experiments on cell adhesion, cell penetration, and investigating the effect of the cell adhesion on the physico-mechanical characteristics of the ES films.

### 3.4. In Vitro Experiments

#### 3.4.1. Cell Seeding Efficiency

**Figure 4:**
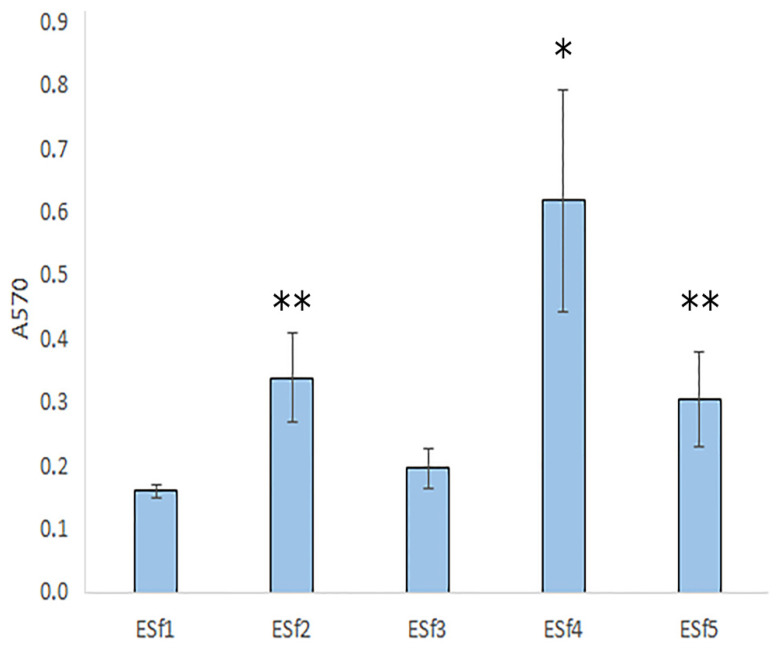
MTT assay results of UC-MSC adhesion on different scaffolds at 48 h after cell seeding. *—significant difference in cell adhesion between the **ESf4** scaffold and all other scaffolds (*p* < 0.05). **—significant differences in cell adhesion between the **ESf2** or **ESf5** scaffold and **ESf1** scaffold (*p* < 0.05).

Cell seeding efficiency was assessed by the colorimetric MTT assay based on the ability of metabolically active cells to convert the water-soluble yellow 3-(4,5-dimethylthiazol-2-yl)-2,5-diphenyltetrazolium bromide (MTT) into insoluble purple intracellular crystals of MTT-formazan. The conversion efficiency is indicative of the general level of dehydrogenase activity of the cells under study, which is, to a certain extent, directly proportional to the concentration of viable cells [67]. The results of the MTT assay show that the **ESf4** scaffold demonstrated the best cell adhesive properties (Figure 4). The sample **ESf5** containing an increased amount of Gt showed a lower ability for cell adhesion in comparison with **ESf4**; a similar pattern was observed for **PCL1**-based scaffolds **ESf2** and **ESf3**. Apparently, the cell adhesive properties of the ES scaffolds correlate with the homogeneity of the fibers’ morphology; it is the PCL/Gt ratio of 80:20 that leads to the absence of structural defects in ES fibers (Figure 4a,c).

#### 3.4.2. Cell–Material Interaction

**Figure 5:**
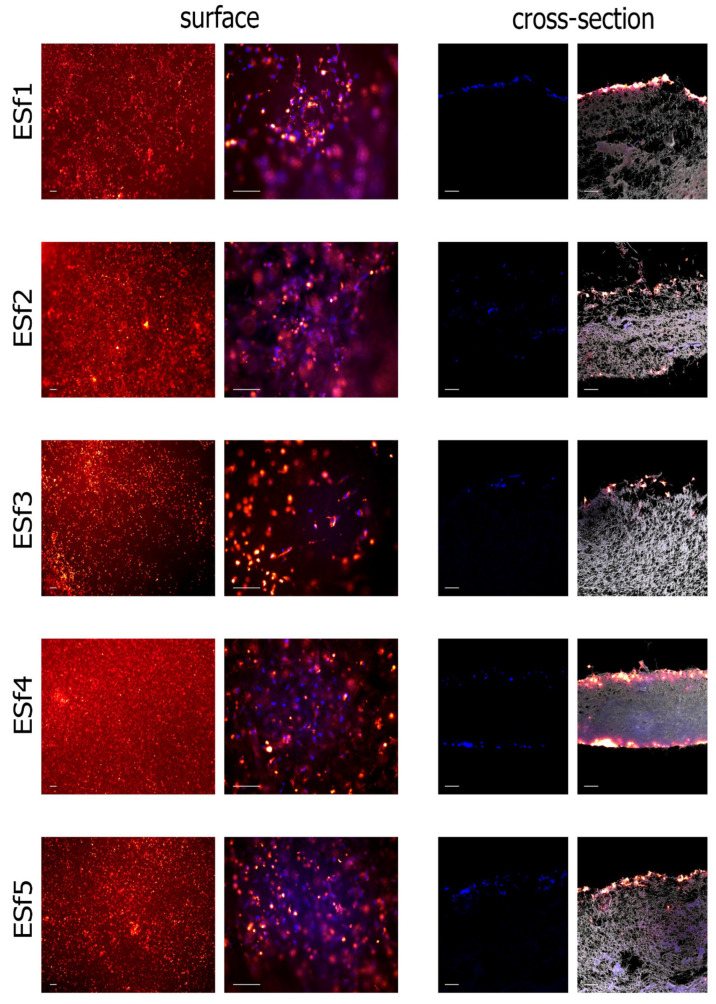
Cell–material interaction after 48 h seeding. MSCs are labeled with PKH26 fluorescent dye (orange), while cell nuclei are counterstained with Hoechst 33,342 (blue). Fluorescent microscopy of cells on the scaffold surface, combined dark field, and fluorescence microscopy of cells on cross sections; scale bar 100 µm.

Cell adhesion for **ESf1**–**ESf5** was studied using the dynamic cell seeding method (see Section 2.6.3). To observe the cell morphology, UC-MSCs were labeled with a fluorescent red-orange vital dye PKH26. All tested scaffolds had good biocompatibility: cells were evenly distributed throughout scaffold surfaces and preserved their fibroblast-like morphology typical for MSCs (Figure 5, left). Combined dark field and fluorescence microscopy of cross cryo-sections showed that cells did not penetrate deep into the thickness of the scaffolds (Figure 5, right).

Summarizing the results of the in vitro experiments, it can be concluded that **PCL2**-based ES films **ESf4** and **ESf5** have several advantages over PCL/Gt ES films prepared with the use of non-functionalized **PCL1**. High biocompatibility makes these materials very promising candidates for biomedical applications. Immersion in physiological media, in combination with cell penetration, could seriously affect the mechanical properties of the scaffolds, thus we decided to clarify this issue.

### 3.5. Mechanical Properties of ES Films

The tensile strength properties of films **ESf1**–**ESf5** were examined at 25 °C using the standard method (see Section 2.2) and the results are illustrated in Table 1. The seeding of ES film prepared from PCL (Table 1, Entry **ESf1**) with UC MSCs had no effect on the mechanical properties of the scaffold. However, Gt containing samples **ESf2**–**ESf5** showed apparent changes in mechanical characteristics. So, in particular, in comparison with **ESf1**, the values of the Young’s modulus before cell seeding were about the same for **ESf2** and **ESf3** and higher for **ESf4** and **ESf5**. After cell seeding, the Young’s modulus remained unchanged for **ESf1** and decreased significantly for composite films **ESf2**–**ESf5**. The cell seeding had a much more explicit effect on the values of elongation at break (ε_p_) values: no effect for **ESf1** and a manifold increase for Gt-containing films **ESf2**–**ESf5**. Note that this effect was most obvious for **PCL2**-based samples **ESf4** and **ESf5**. We assume that this effect occurs as a result of more uniform distribution of PCL and Gt macromolecules in the composite when using NHS-functionalized **PCL2**, which leads to a more pronounced plastification effect of water arising from the interaction of Gt with aqueous media. Scaffolds with similar mechanical properties can be used for engineering of artificial elastic tissues, for example, tendons.

It should also be noted that Gt-cross-linked fibrous mats with 30 wt% Gt content demonstrated a higher elongation at break (ε_p_) value in comparison with PCL-based material [30], whereas **ESf5** had a lower ε_p_ in comparison with **ESf1**.

## 4. Conclusions

In summary, in the present work, we tried to assess the potential of reactive functionalized polyesters in the preparation of composite ES scaffolds, containing covalently bonded gelatin. Previously known approaches to similar materials are based on grafting or cross-linking *after* ES molding. We proposed that NHS-functionalized **PCL2** can react with gelatin directly in spinning solution, and have proved our assumption experimentally. Our study led to the following generalized conclusions:

- On the example of **PCL2**, it is determined that chain-end NHS functionalization of the polyester is quite sufficient to provide polyester-Gt covalent binding.
- The reaction between gelatin and **PCL2** can be conducted in HFIP spinning solution. However, as shown on the model reaction of **NHS-OMe** with *n*-BuNH_2_, corresponding amide and HFIP ester are formed simultaneously. Fortunately, esterification can be inhibited by minimal amounts of AcOH.
- The use of **PCL2**/Gt/HFIP/AcOH spinning solution provides excellent morphology of the ES fibers, reminiscent of the extracellular matrix.
- Hydrolytic degradation experiments have demonstrated that **PCL2**/Gt ES fibers remain stable in composition in aq.dist, 0.1 M aq. PBS, and 0.1 M aq. NaHCO_3_, even for 14-day exposition.
- Electrospun polymer scaffolds, prepared with the use of **PCL2**, have demonstrated higher values of cell adhesion and cell penetration by an example of UC MSCs.
- Cell adhesion and cell penetration have a significant impact on the mechanical properties of **PCL2**-based ES films, vastly increasing their elasticity.

Comparison of **PCL2**/Gt-based ES fiber materials to PCL/Gt composites prepared with the use of [PCL ES]→[PCL grafting]→[Gt binding] [22,23,24,44,52] and [PCL ES]→[Gt cross-linking] [26,28,29,30,40,42,42,45,55] shows the following with regard to the most important characteristics of ES films: **PCL2**/Gt-based ES fiber demonstrates higher hydrolytic stability and retains the integrity of the structure longer; more importantly, the mechanical characteristics of **PCL2**/Gt-based ES scaffolds differ significantly from the characteristics of Gt-grafted or Gt-cross-linked PCL-based scaffolds.

In this way, chain-end modification of the polyester macromolecules by reactive groups seems to be a realistic method that is too good to be left out in developing formulations for subsequent ES molding. However, when designing specific formulations of the complex ES solutions, it is important to pay attention to ensure that possible side reactions with the participation of the ES solvent is avoided.

## Figures and Tables

**Table 1:** Mechanical properties of ES mats ^1^.

Entry	Gt wt%	Young’s Modulus before Cell Seeding, N·mm^−2^	Young’s Modulus after Cell Seeding, N·mm^−2^	Elongation at Break ε_p_, before Cell Seeding, %	Elongation at Break ε_p_, after Cell Seeding, %
**ESf1**	0	22.8 ± 12.3	21.1 ± 4.9	27.3 ± 20.9	29.7 ± 15.2
**ESf2**	20	20.7 ± 15.5	12.2 ± 8.9	34.9 ± 13.6	185.9 ± 37.5
**ESf3**	30	39.6 ± 19.1	25.5 ± 9.4	20.3 ± 4.0	111.5 ± 18.1
**ESf4**	20	57.9 ± 16.0	34.3 ± 6.3	8.3 ± 4.7	464.3 ± 36.7
**ESf5**	30	60.4 ± 20.0	26.1 ± 6.9	5.8 ± 1.3	752.8 ± 200.6

^1^ The thickness of all ES films before and after cell seeding was 0.5 mm.

## Data Availability

The data presented in this study are available upon request from the corresponding author.

## Supplementary Materials

The following are available online at https://www.mdpi.com/article/10.3390/polym14194203/s1, the synthesis of **NHS-OMe**, Figure S1: ^1^H NMR spectrum (400 MHz, CDCl_3_, 20 °C) of **NHS-OMe**; Figure S2: ^13^C {^1^H} NMR spectrum (101 MHz, CDCl_3_, 20 °C) of **NHS-OMe**; Figure S3: ^1^H NMR spectrum (400 MHz, CDCl_3_, 20 °C) of **PCL1**; Figure S4: ^1^H NMR spectrum (400 MHz, CDCl_3_, 20 °C) of **PCL2**; Figure S5: Microphotograph of the sample **ESf0**; Figure S6: FT-IR spectra of the sample **ESf0** before and after 7 days of hydrolytic degradation; Figure S7: ^1^H NMR spectrum (400 MHz, CDCl_3_, 20 °C) of the mixture of **NHS-OMe**, HFIP, and Et_3_N (the reaction time 1 min); Figure S8: ^1^H NMR spectrum (400 MHz, CDCl_3_, 20 °C) of the mixture of **NHS-OMe**, HFIP, and pyridine (the reaction time 3 h); Figure S9: ^1^H NMR spectrum (400 MHz, CDCl_3_, 20 °C) of the mixture of **NHS-OMe**, HFIP, gelatin, and AcOH (reaction time of 1 d); Figure S10: ^1^H NMR spectrum (400 MHz, CDCl_3_, 20 °C) of the mixture of **NHS-OMe**, HFIP, gelatin, and AcOH (the reaction time 30 d); Figure S11: Microphotograph of the sample **ESf6**; Figure S12: FT-IR spectra of the sample **ESf6** before and after 3 days of hydrolytic degradation.
