# Supplementary material for: Chain-End Functionalization of Poly(ε-caprolactone) for Chemical Binding with Gelatin: Binary Electrospun Scaffolds with Improved Physico-Mechanical Characteristics and Cell Adhesive Properties
Source: Polymers (Basel). 2022 Oct 7;14(19):4203. doi: 10.3390/polym14194203 (PMC9570970; doi:10.3390/polym14194203)
Supplement: Supplementary file 1 [file polymers-14-04203-s001.zip › polymers-1941655-supplementary.pdf]

Chain-End Functionalization of Poly(ε-caprolactone) for Chemical Binding with Gelatin: Binary Electrospun Scaffolds with Improved Physico-Mechanical Characteristics and Cell Adhesive Properties

Ilya Nifant'ev [1,2,3], Victoria Besprozvannykh [1,3], Andrey Shlyakhtin [1,2], Alexander Tavtorkin [1], Sergei Legkov [1], Maria Chinova [1], Irina Arutyunyan [4,5], Anna Soboleva [5,6], Timur Fatkhudinov [5,6] and Pavel Ivchenko [1,2,*]

[1] A.V. Topchiev Institute of Petrochemical Synthesis RAS, 29 Leninsky Pr., 119991 Moscow, Russia
[2] Chemistry Department, M.V. Lomonosov Moscow State University, 1–3 Leninskie Gory, 119991 Moscow, Russia
[3] Chemistry Department, National Research University Higher School of Economics, 20 Miasnitskaya Street, 101000 Moscow, Russia
[4] Research Center for Obstetrics, Gynecology and Perinatology, Ministry of Healthcare of the Russian Federation, 4 Oparin Street, 117997 Moscow, Russia
[5] Institute of Medicine, Peoples' Friendship University of Russia, Miklukho-Maklaya 6 Street, 117198 Moscow, Russia

[6] Research Institute of Human Morphology, 3 Tsyurupy Street, 117418 Moscow, Russia

* Correspondence: phpasha1@yandex.ru; Tel.: +7-495-939-4098

Supplementary Information

S1. Synthesis and NMR spectra of **NHS-OMe** S2

S2. NMR spectra of the polymers S3

S3. Preliminary ES experiments S4

S4. Reactivity of **NHS-OMe** in the model spinning solution S5

S5. Morphology and degradation of **ESf6** S7

S1. Synthesis and NMR spectra of NHS-OMe

The solution of DMAP (1.85 g, 15.2 mmol) in CH_2Cl_2 (10 mL) was added dropwise within 30 min to the solution of **NHS-Cl** (3.76 g, 15.2 mmol, 1 eq.) and methanol (614 µL, 15.2 mmol, 1 eq.) in CH_2Cl_2 (25 mL) at 5 °C. The mixture was stirred at room temperature for 30 min, washed with 1M HCl, with water, dried over $MgSO_4$ and evaporated to give residue oil, which was treated by hexane (10 mL) to give white solid, m.p. = 38 °C. The yield was 2.48 g (67 %). ^1H and ^{13}C {^1H} NMR spectra of **NHS-OMe** are presented in Figures S1 and S2, respectively.

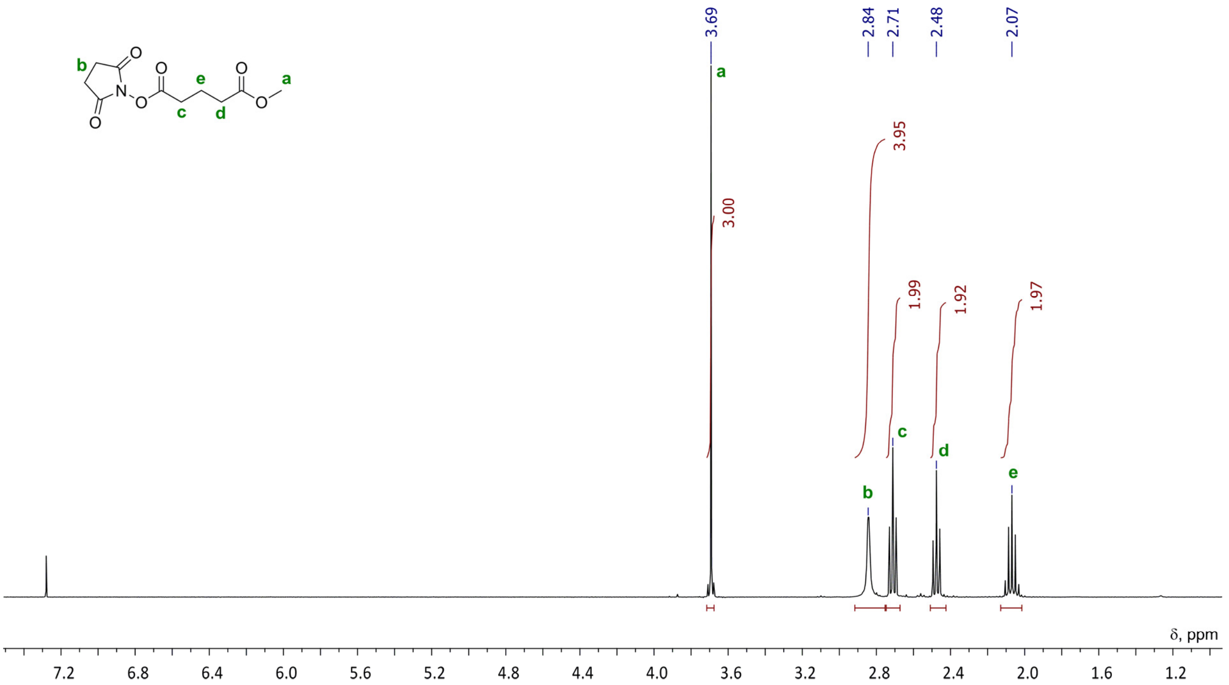

Figure S1. ^1H NMR spectrum (400 MHz, $CDCl_3$, 20 °C) of **NHS-OMe**.

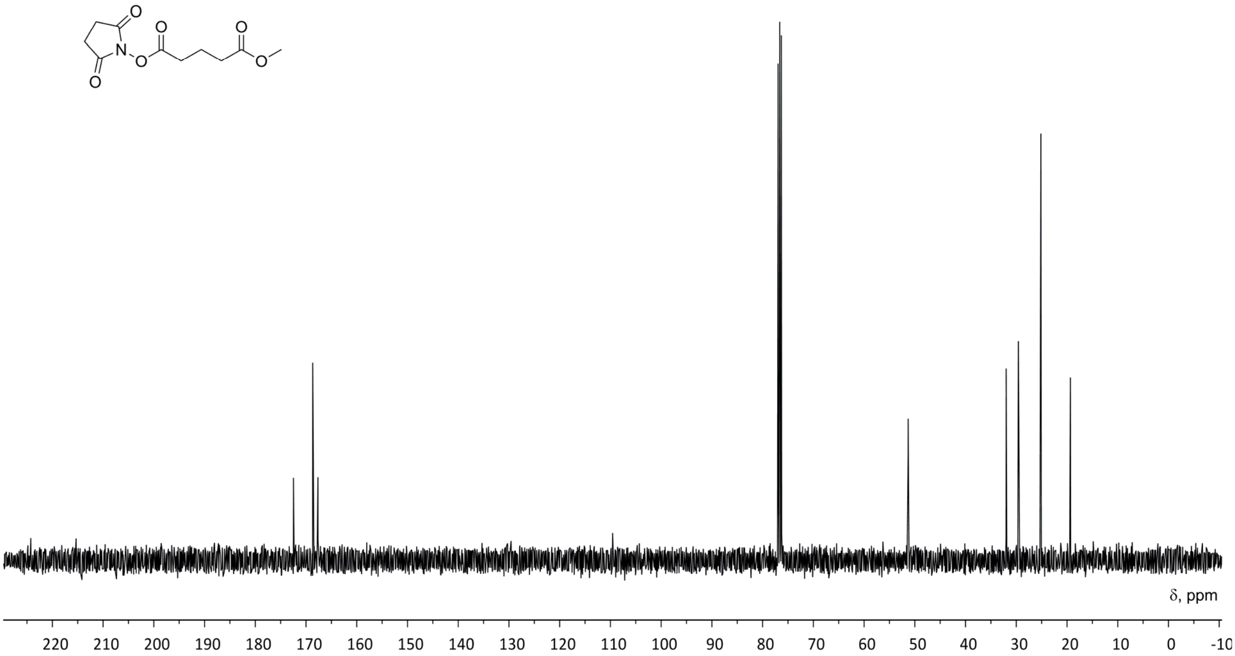

Figure S2. ^{13}C {^1H} NMR spectrum (101 MHz, $CDCl_3$, 20 °C) of **NHS-OMe**.

S2. NMR spectra of the polymers

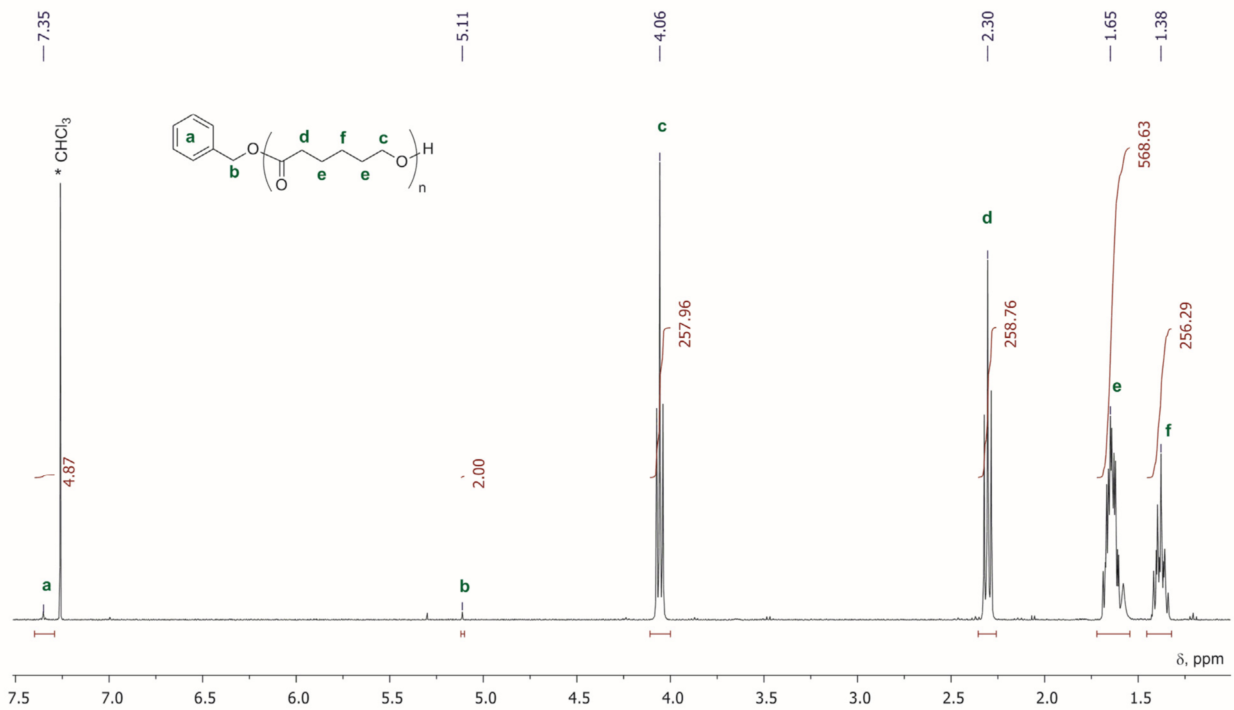

Figure S3. ^{1}H NMR spectrum (400 MHz, $CDCl_3$, 20 °C) of **PCL1**.

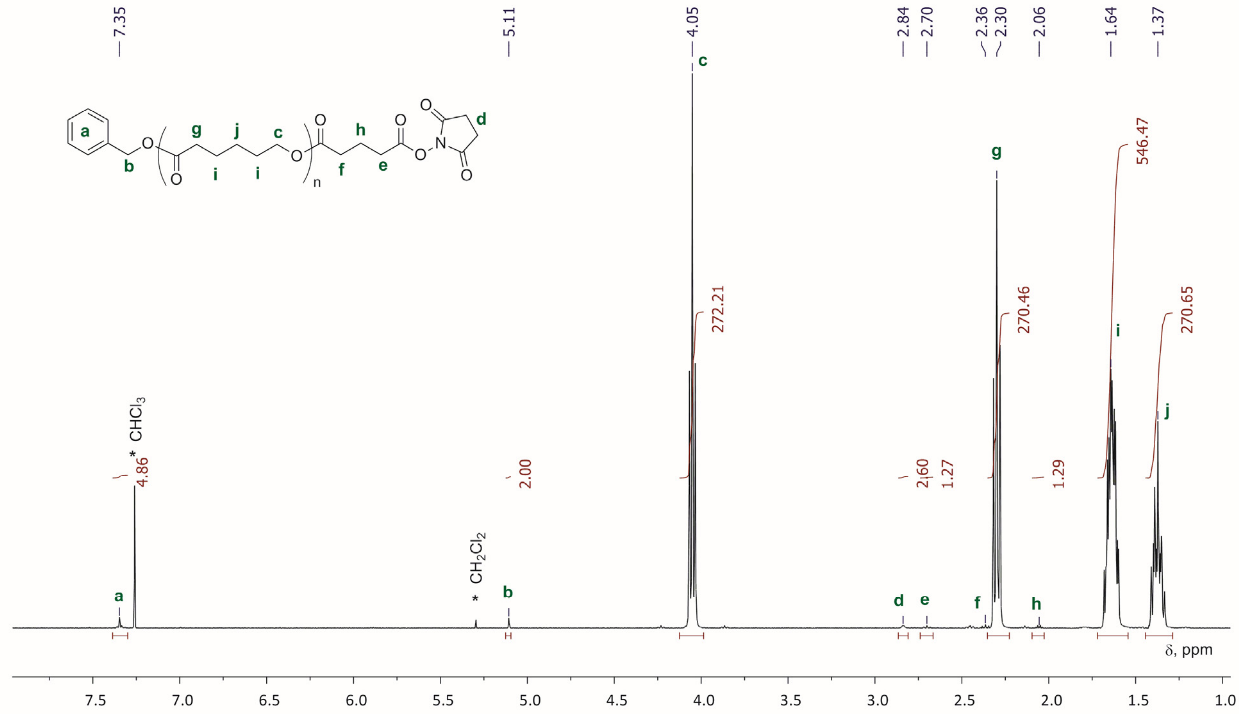

Figure S4. ^{1}H NMR spectrum (400 MHz, $CDCl_3$, 20 °C) of **PCL2**.

S3. Preliminary ES experiments

Preliminary ES experiments were aimed to achieve suitable and homogeneous morphology of the ES mats with the use of PCL/Gt mixtures. This task was solved by the use of HFIP as a solvent and by optimization of the ES molding parameters. Microphotograph of the sample **ESf0** (70 wt% of **PCL2** and 30 wt% of Gt) is presented in Figure S5.

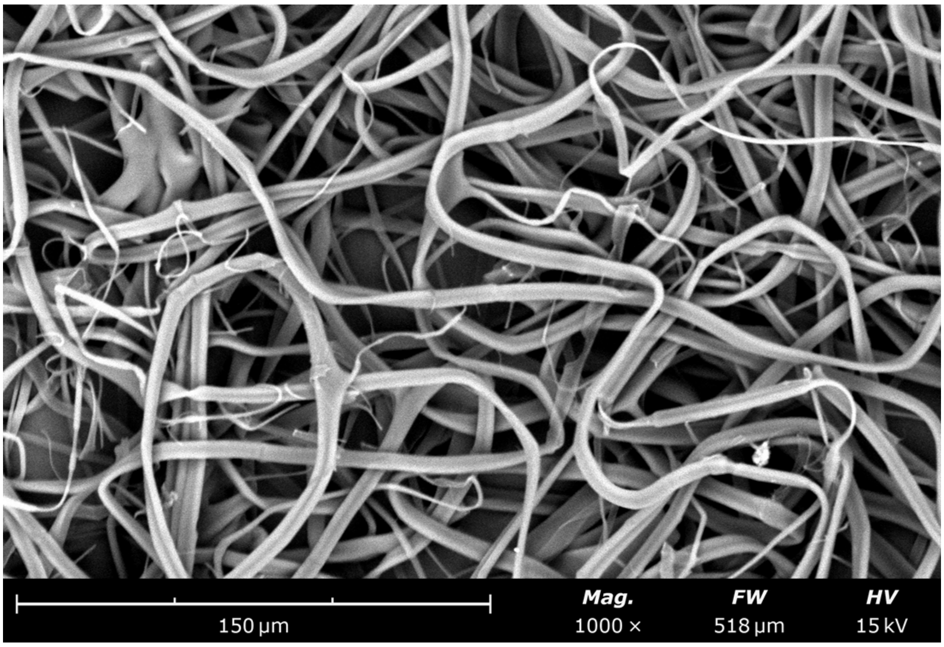

Figure S5. Microphotograph of the sample **ESf0**.

As can be seen in Figure S5, ES material had generally homogeneous morphology, but a certain amount of thin filaments was formed. However, hydrolytic stability is no less important than morphology for the possibilities of biomedical using ES fibrous mats, and we studied hydrolytic stability of the sample **ESf0** in three environments (H_2O, 0.1M PBS and 0.1M $NaHCO_3$ aq. solutions). As can be seen in Figure S06, substantial part of Gt was washed away after 1 week.

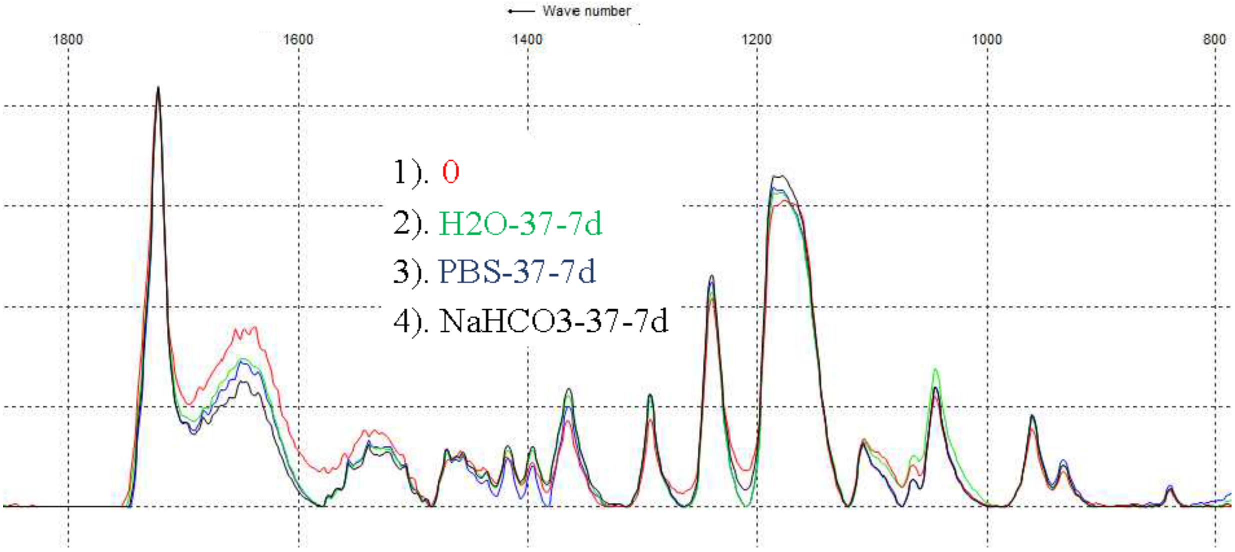

Figure S6. FT-IR spectra of the sample **ESf0** before and after 7-days hydrolytic degradation.

S4. Reactivity of NHS-OMe in the model spinning solution

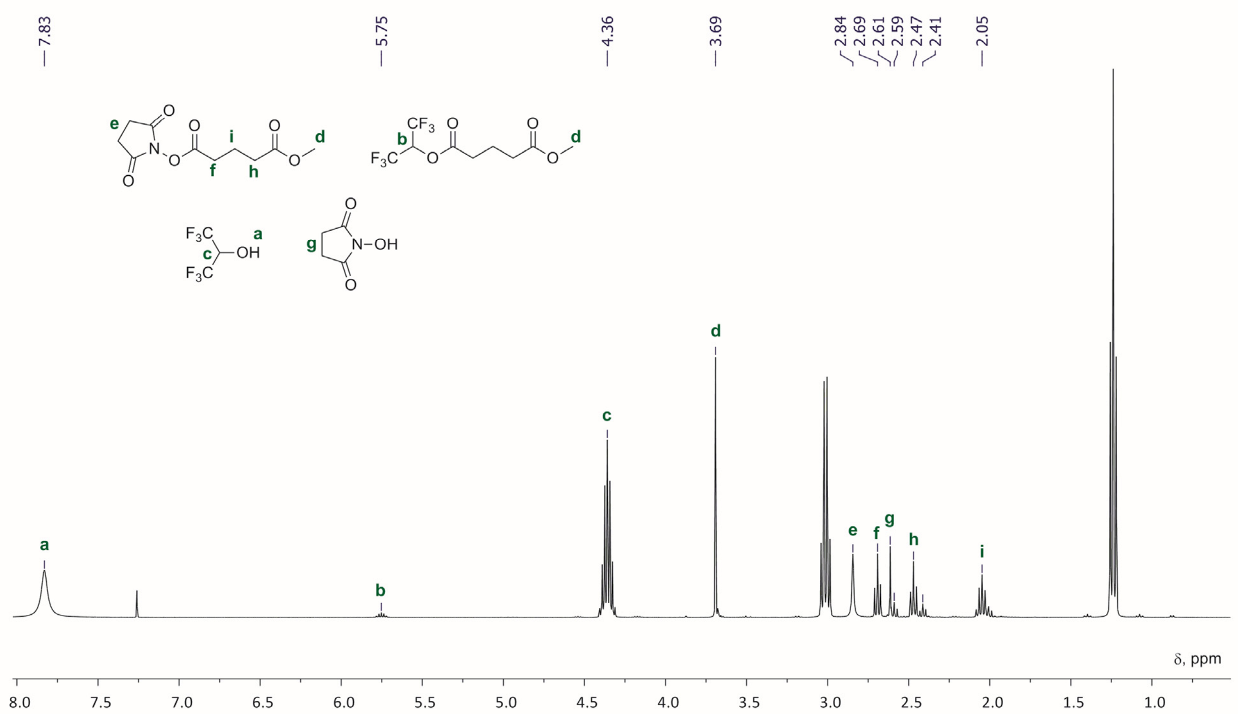

Figure S7. ^{1}H NMR spectrum (400 MHz, $CDCl_3$, 20 °C) of the mixture of **NHS-OMe**, HFIP and Et_3N (the reaction time 1 min).

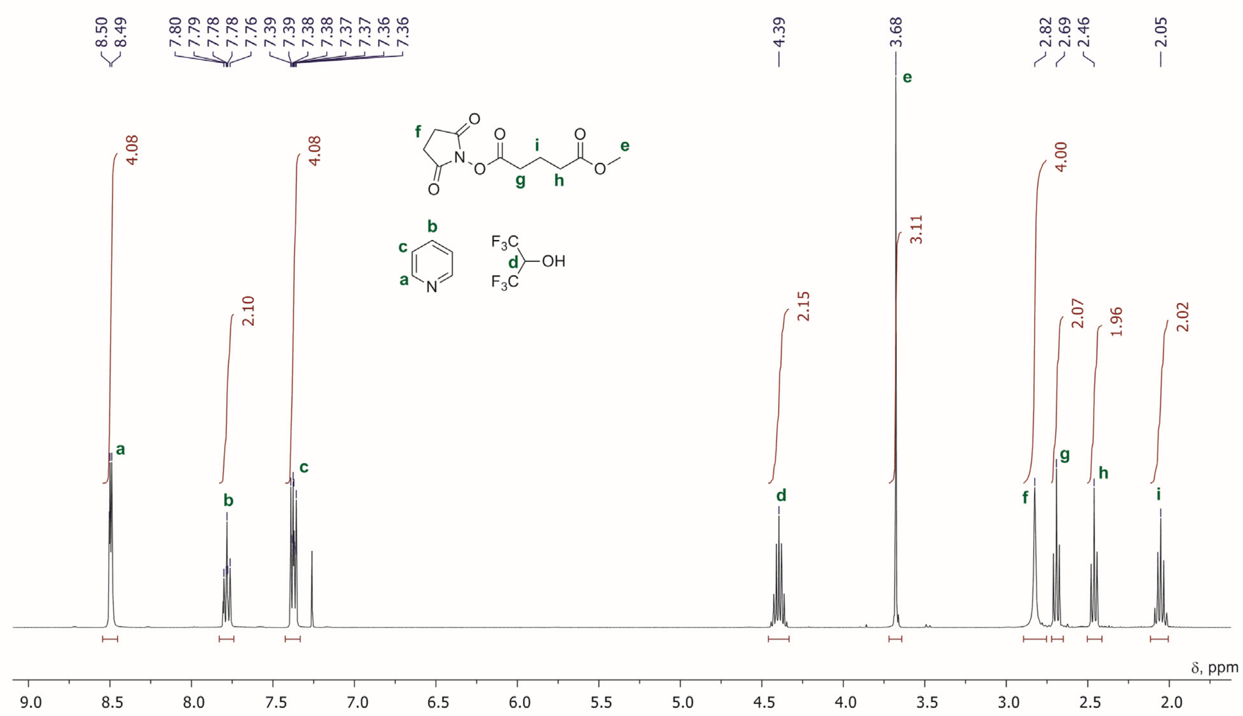

Figure S8. ^{1}H NMR spectrum (400 MHz, $CDCl_3$, 20 °C) of the mixture of **NHS-OMe**, HFIP and pyridine (the reaction time 3 h).

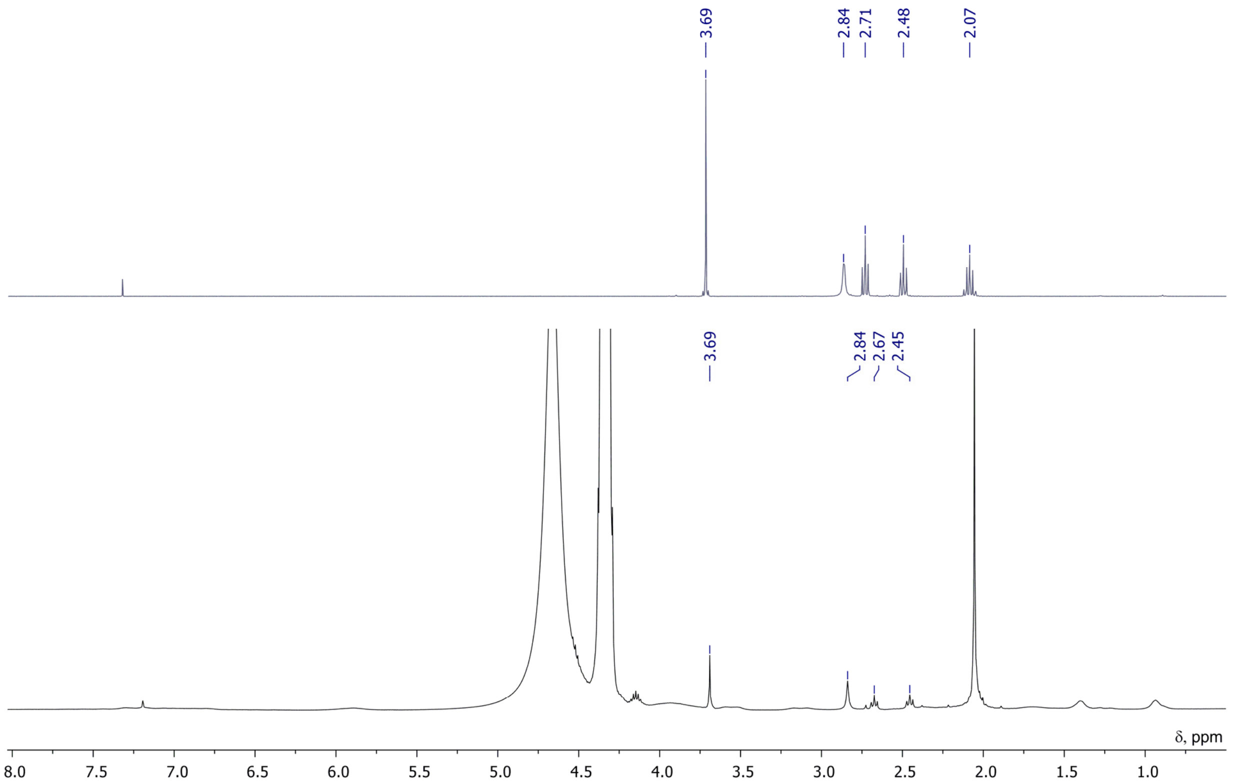

Figure S9. ¹H NMR spectrum (400 MHz, $CDCl_3$, 20 °C) of the mixture of **NHS-OMe**, HFIP, gelatin and AcOH (the reaction time 1 d).

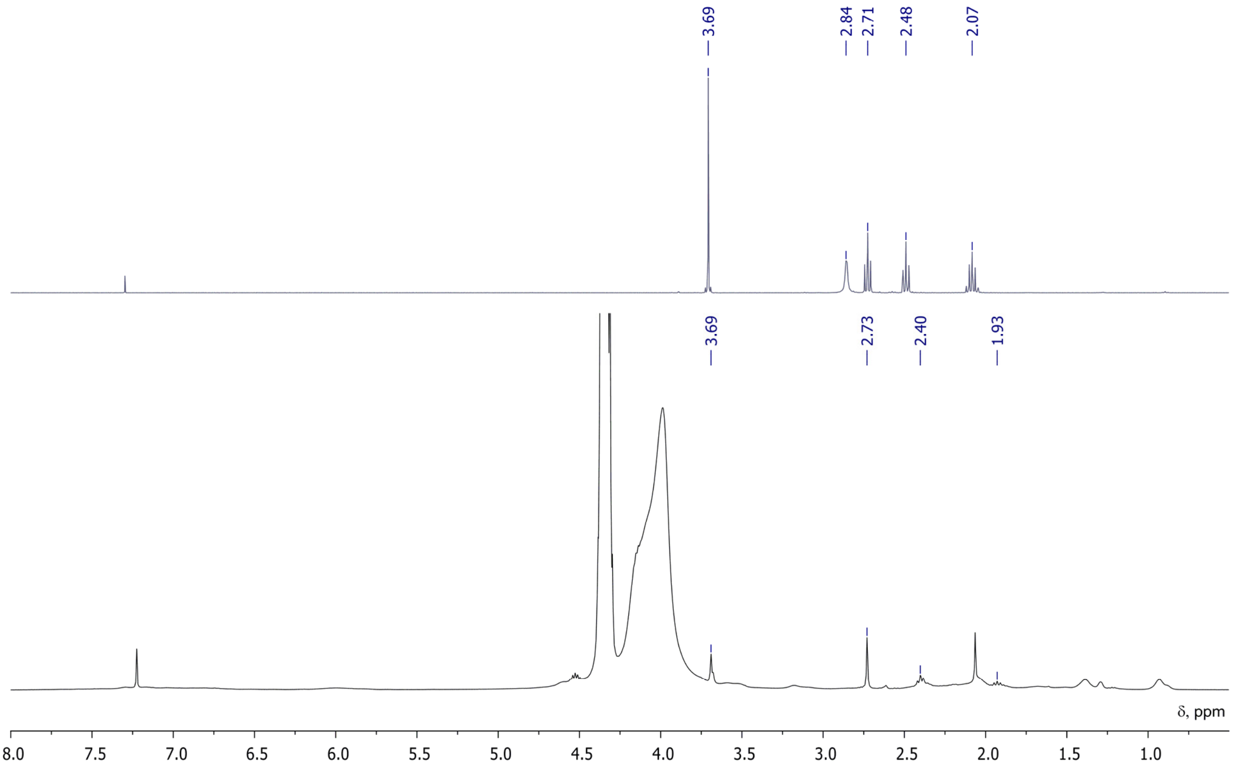

Figure S10. ¹H NMR spectrum (400 MHz, $CDCl_3$, 20 °C) of the mixture of **NHS-OMe**, HFIP, gelatin and AcOH (the reaction time 30 d).

S5. Morphology and degradation of ESf6

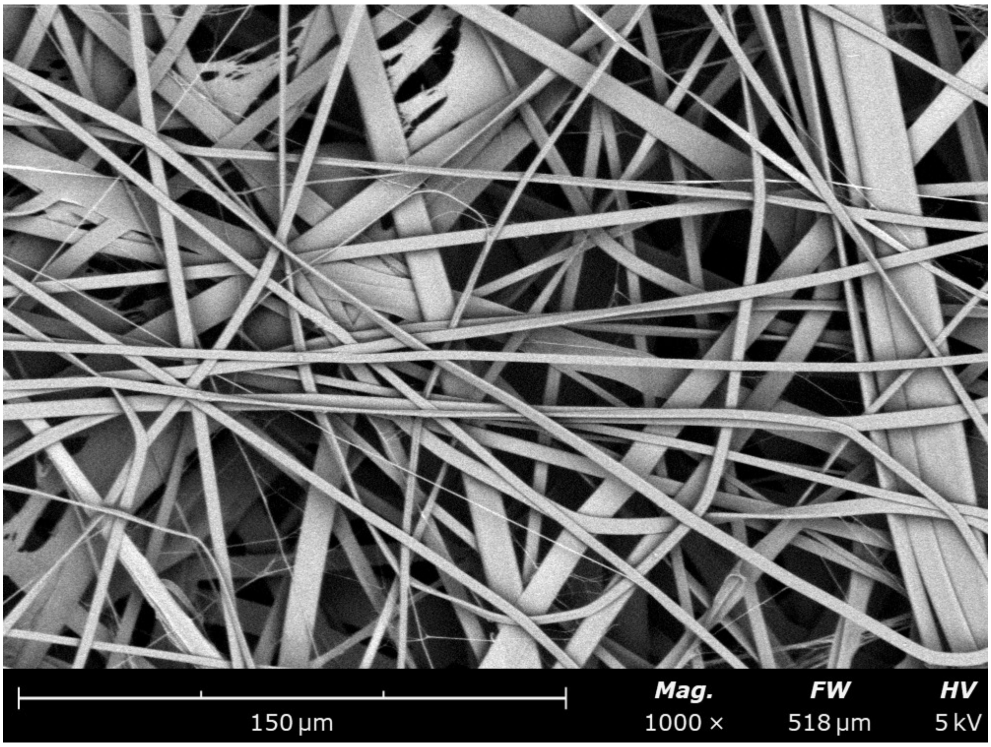

Figure S11. Microphotograph of the sample **ESf6**.

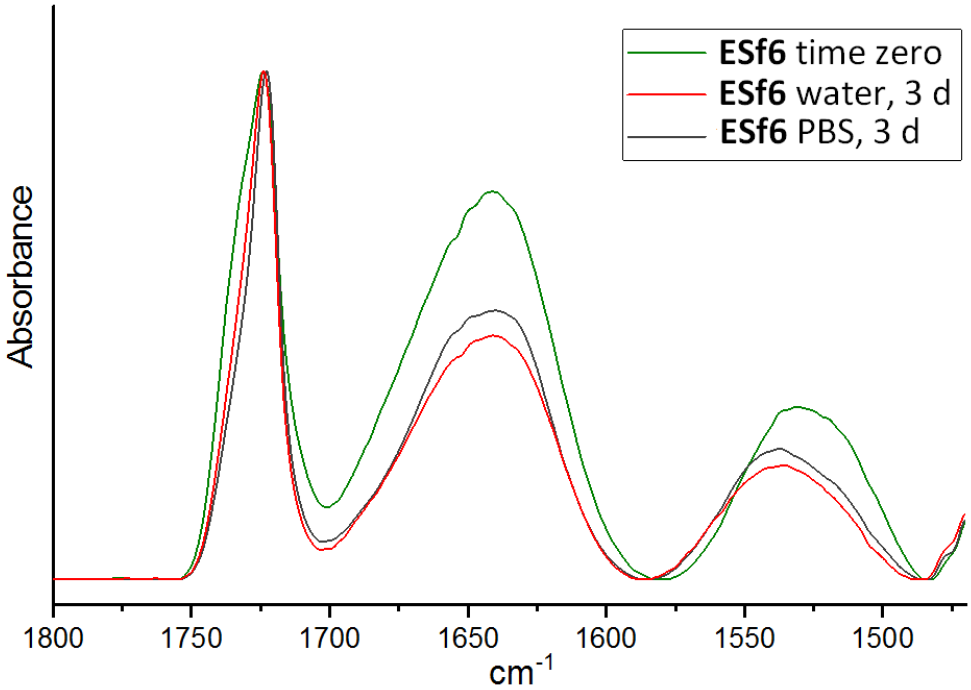

Figure S12. FT-IR spectra of the sample **ESf6** before and after 3-day hydrolytic degradation.
